# Feasibility of a computer-assisted alcohol SBIRT program in an urban emergency department: patient and research staff perspectives

**DOI:** 10.1186/1940-0640-8-2

**Published:** 2013-01-16

**Authors:** Mary K Murphy, Polly E Bijur, David Rosenbloom, Steven L Bernstein, E John Gallagher

**Affiliations:** 1 Department of Emergency Medicine, Yale University School of Medicine, 464 Congress Ave, Suite 260, 06519, New Haven, CT, USA; 2 Department of Emergency Medicine, Albert Einstein College of Medicine, Bronx, NY, USA; 3 Boston University School of Public Health, Join Together, Boston, MA, USA

**Keywords:** Computerized alcohol screening, Brief intervention, Emergency department, SBIRT

## Abstract

**Objectives:**

The study objective was to assess the feasibility of a computerized alcohol-screening interview (CASI) program to identify at-risk alcohol users among adult emergency department (ED) patients. The study aimed to evaluate the feasibility of implementing a computerized screening, brief intervention, and referral to treatment (SBIRT) program within a busy urban ED setting, to report on accurate deployment of alcohol screening results, and to assess comprehension and satisfaction with CASI from both patient and research staff perspectives.

**Methods:**

Research assistants (RAs) screened a convenience sample of medically stable ED patients. The RAs brought CASI to patients’ bedsides, and patients entered their own alcohol consumption data. The CASI intervention consisted of an alcohol use screening identification test, a personalized normative feedback profile, NIAAA low-risk drinking educational materials, and treatment referrals (when indicated).

**Results:**

Five hundred seventeen patients were enrolled. The median age of participants was 37 years (range, 21-85 years); 37% were men, 62% were Hispanic, 7% were Caucasian, 30% were African American, and 2% were multiracial. Eighty percent reported regular use of computers at home. Eighty percent of patients approached consented to participate, and 99% of those who started CASI were able to complete it. Two percent of interviews were interrupted for medical tests and procedures, however, no patients required breaks from using CASI for not feeling well. The CASI program accurately provided alcohol risk education to patients 100% of the time. Thirty-two percent of patients in the sample screened positive for at-risk drinking. Sixty percent of patients reported that CASI increased their knowledge of safe drinking limits, 39% reported some likeliness to change their alcohol use, and 28% reported some intention to consult a health care professional about their alcohol use as a result of their screening results. Ninety-three percent reported CASI was easy to use, 93% felt comfortable receiving alcohol education via computer, and 89% liked using CASI. Ninety percent of patients correctly identified their alcohol risk level after participating in CASI. With regard to research staff experience, RAs needed to provide standby assistance to patients during <1% of CASI administrations and needed to troubleshoot computer issues in 4% of interviews. The RAs distributed the correct alcohol risk normative profiles to patients 97% of the time and provided patients with treatment referrals when indicated 81% of the time. The RAs rated patients as “not bothered at all” by using CASI 94% of the time.

**Conclusions:**

This study demonstrates that an ED-based computerized alcohol screening program is both acceptable to patients and effective in educating patients about their alcohol risk level. Additionally, this study demonstrates that few logistical problems related to using computers for these interventions were experienced by research staff: in most cases, staff accurately deployed alcohol risk education to patients, and in all cases, the computer provided accurate education to patients. Computer-assisted SBIRT may represent a significant time-saving measure, allowing EDs to reach larger numbers of patients for alcohol intervention without causing undue clinical burden or interruptions to clinical care. Future studies with follow-up are needed to replicate these results and assess drinking reductions post-intervention.

## Introduction

Alcohol misuse is the third leading cause of preventable death in the United States today [1]. The costs to society amount to over 220 billion dollars annually [2]. Most at-risk drinkers never receive needed alcohol treatment services [3]; however, each year over 130 million people visit an emergency department (ED), creating an opportunity to screen a large number of people who may otherwise have never been asked about their alcohol use or offered alcohol screening, brief intervention, and referral for treatment (SBIRT). Prior research shows that there is a high prevalence of unmet substance abuse treatment need among adult ED patients in general [4]; estimates show that as many as 46% of ED patients [5] have recently consumed alcohol, and a significant number of the 31.6 million injury-related ED visits in the US are alcohol-related [6].

The role of the ED is often to aid in the identification of patients who are at-risk or dependent drinkers who require education and/or referral to specialty treatment to avoid the deleterious effects of alcohol. Several prior alcohol SBIRT studies have demonstrated positive results in the ED and in primary-care settings over the last two decades [7], showing reductions in consumption and consequences of excessive use [8,9]. However the evidence has been mixed [10], leaving important questions regarding how best to reach more people who would benefit from alcohol SBIRT without interrupting clinical care, increasing staff burden or cost, or sacrificing effectiveness.

Considering the large numbers of patients seen in the ED every day, widespread or universal screening for alcohol misuse among ED patients is a challenging goal. Barriers to wider adoption of face-to-face SBIRT have included cost, staff time, and training [11-14]. A computer-assisted model of SBIRT might make it possible to minimize such barriers. Recent studies have explored the use of computers to help increase the number of patients who can be reached for alcohol misuse, with some promising results [13,15]. Integrating computer-based SBIRT into ED clinical practice, however, is not without challenges. Nilsen and colleagues [16] recently reported on the implementation of a computerized alcohol feedback program in their ED that utilized a waiting-room kiosk model and found several logistical barriers and low overall participation rates. However, Neumann et al [17] recently published a randomized clinical trial of 1139 risky drinkers who received computerized tailored brief advice during a single ED visit with no intervention boosters and found high patient acceptability and a significant decrease in alcohol use at 6 and 12 month follow-up: computer intervention patients, compared with controls, decreased alcohol consumption by 36% versus 20% (p = 0.006) respectively at six months, and by 23% versus 11% (p = 0.02) respectively at 12 months.

Since adoption of computer-assisted SBIRT has been slow, it is important to carefully evaluate and report on the feasibility of its implementation into ED practice in order to disseminate best practices for implementation of similar programs in other EDs. In this study, we report on our experiences deploying computer-assisted SBIRT and evaluate the feasibility of its implementation, taking into account patient and research staff experiences. To our knowledge, no study to date has evaluated the feasibility of a computer-assisted ED SBIRT program for alcohol use including feedback from research staff. The study had two related goals: 1) to assess the feasibility of using a computerized alcohol screening interview (CASI) program to identify at-risk alcohol users among adult ED patients and 2) report on the accurate deployment, or distribution, of alcohol screening results from both patient and research staff perspectives.

## Methods

### Study design

The current study used a cross-sectional design to survey a convenience sample of adult ED patients who completed an alcohol screening and educational intervention computer program during a routine ED visit (the CASI program). Patients identified by CASI as at-risk drinkers were provided referrals to treatment. All patients, regardless of alcohol risk level, were also provided with written National Institute on Alcohol Abuse and Alcoholism (NIAAA) educational materials as well as a national treatment locator info-line phone number to take home. Patients were all given the option to complete their paper and computerized assessments in English or Spanish. Informed consent and all study procedures were approved by the hospital’s Institutional Review Board prior to study commencement.

### Study setting

The study was conducted in the adult section of Montefiore Medical Center, the academic medical center of the Albert Einstein College of Medicine in the Bronx, New York. This ED, the second largest in the US, is a level-2 trauma center serving over 100,000 patients annually from a diverse community of over 2 million residents.

### Study population

#### Patient participants

The demographic breakdown of the ED population reflects the diverse community it serves: approximately 60% were Hispanic (inclusive of Caucasian/white and African American descent), 25% were African-American, 10% were Caucasian/ white, and the remaining 5% were Asian or Native American, or did not provide race/ethnicity data.

#### Research staff participants

Five research assistants (RAs) assisted in this study and completed surveys recording their experiences enrolling each patient. All RAs were bilingual (English/Spanish) and of similar race and ethnicity to that of the study population. Research assistants working in the Montefiore Medical Center ED are all qualified technicians, assisting nurses when needed but primarily implementing research protocols. They staff the ED 24 hours per day, seven days per week, and are salaried employees. All RAs who assisted in this study were seasoned ED staff with a median length of employment of five years.

### Study protocol

The RAs approached a convenience sample of adult patients entering the ED if they were medically stable, alert and oriented, spoke English or Spanish, were in the ED for greater than one hour (less acute phase of treatment, and therefore less likely to interrupt clinical care) to determine study eligibility and obtain informed consent. Patients presenting to the ED for a primary psychiatric complaint were not eligible because they were triaged and sent to a separate psychiatric observation unit. Similarly, patients aged ≤ 20 years were not included because they were triaged to a separate pediatric ED. Participants were enrolled seven days a week, 24 hours a day, when RAs were available during a three-month period. Between other RA responsibilities and uncovered shifts, this equated to RAs enrolling approximately three patients per day. Patients were not compensated for their participation.

The RAs explained to eligible patients that they were being asked to participate in an anonymous, brief, computer-assisted alcohol screening research project. After RAs completed verbal consent procedures, they wheeled the CASI mobile cart (a tablet computer attached to an IV pole, designed to blend into the ED environment as standard medical equipment) to each patient’s stretcher or treatment room and offered noise-cancelling headphones to drown out ED distractions. The RAs remained nearby to be available for patient questions when necessary, but to ensure privacy, they stood within earshot but not over the patient’s bedside. Patients entered their data directly into the CASI computer via the computer, which recorded their data anonymously (ensured by staff-provided ID numbers) and privately, while they waited for treatment in various clinical areas of the ED.

The first screen of the CASI program served as a computer screening test for patients as well as a brief orientation to the computer, collecting brief demographics (e.g., age and sex, which enabled CASI to generate normative alcohol educational feedback). These tasks required the patient to be able to interact with the computer mouse, enter text using the keyboard, and use a stylus to enter data via drop-down menus. Though it was never exercised, RAs had discretion to stop study entry procedures and list the patient as ineligible for computer literacy reasons if the patient experienced significant difficulty entering data independently.

When each patient were done entering his or her data, the RA returned and asked the patient to complete a brief acceptance and comprehension paper-and-pencil questionnaire (see measures). Research staff then completed their own paper and pencil questionnaire to record their experience implementing CASI. After all procedures were complete, RAs provided each patient with a confidential sealed envelope containing written NIAAA low-risk drinking educational materials and a list of local alcohol treatment referral sources (prepared in advance by author MM). the RAs also provided patients with a copy of the customized normative alcohol risk profile generated by CASI.

## Measures

### Demographics

The RAs recorded age, sex, race, ethnicity, primary language, education, level of prior computer experience, triage diagnosis, and language version (English or Spanish). This information was recorded on a paper-and-pencil data collection instrument not connected to the CASI program to ensure patient anonymity. The remainder and majority of all data were collected via computer. As we were interested in feasibility of implementing this program, rates and reasons for all study refusals, exclusions, and incomplete CASI administrations were also recorded.

### The CASI computer program

The CASI program was a syndicated version of the website http://www.alcoholscreening.org provided to our study group at no charge by Join Together/Boston University School of Public Health (now owned and operated by The Partnership® at Drugfree.org). The online program, described elsewhere [18,19], has been freely available to the public since 2001, enabling over 200,000 visitors per year to receive research-based, empirically validated education about safe drinking limits based on the Alcohol Use Disorders Identification Test (AUDIT) (described below). Visitors to the site can complete alcohol self-assessments, educate themselves on risky drinking, and seek local treatment referrals when needed based on zip codes (thus receiving all the components of SBIRT). For the purposes of this study, the site contents were translated into Spanish so patients with limited English could complete the study in their native language. Minor tailoring of the website was also completed to collect research IDs on the home screen for data tracking before the study commenced.

Following the initial demographic screen, CASI began with the 10-item AUDIT screening questionnaire. Developed by the World Health Organization, the AUDIT is considered the “gold standard” method of identifying people with hazardous or harmful alcohol use [20]. This assessment tool was developed specifically to help health-care practitioners identify people who would benefit from reducing or stopping their use of alcohol. In this study, we used the generally accepted score of ≥8 as a cutoff for at-risk drinking. Although it is beyond the scope of this paper to outline the full psychometric history of the AUDIT and choice of cutoff scores, we refer the reader to de Meneses-Gaya et al. [21] for a recent systematic review.

The CASI was programmed to allow patients to receive tailored alcohol risk-level education based on their age, sex, and self-reported consumption data. For the purposes of this study, at-risk drinkers (anyone above low-risk) were classified according to NIAAA drinking guidelines, which define low-risk drinking as no more than four drinks per occasion on any single day or 14 drinks per week for men, and no more than three drinks per occasion on any single day or seven drinks per week for women and men over age 65 [22].

The CASI presented alcohol risk levels on the computer screen based on the theme of a traffic light: a green light communicated low risk, a yellow light communicated medium risk, and a red light indicated high risk. Specifically, patients were classified as “low risk” if they reported drinking within NIAAA low-risk drinking guidelines and scored <8 on the AUDIT, “medium risk” if they reported drinking in excess of NIAAA low-risk guidelines but scored <8 on the AUDIT, and “high risk” if they scored ≥8 on the AUDIT regardless of daily or weekly consumption.

The CASI’s final computer screen displayed the patient’s alcohol risk level with normative feedback indicating how their alcohol consumption compared with others of the same age and sex. Patients who scored above low-risk guidelines were given the option to click a button called “Find help now,” which was programmed to allow patients to enter their zip code to access a list of local alcohol treatment agencies. The RAs printed this list for patients to take home with them as well.

### Patient acceptance and comprehension questionnaire

In order to assess patient acceptance of CASI content, patients completed a self-administered paper-and-pencil questionnaire after completing CASI to report their level of satisfaction with different aspects of the computer program. Specifically, patients were asked to provide Likert responses between 1 (not at all) and 5 (very much) for questions such as, “How helpful was this for you?” and “How much did some parts of the computer program bother you?” Additional questions required yes or no responses, e.g., “Did you like using this program?” “Was the program easy to use?” and “Did participating in this program get you thinking about your alcohol use?”.

To assess patient comprehension of their alcohol-risk level, patients were asked to identify from a list of the following three items which alcohol risk feedback level the CASI program assigned to them based on their screening answers: 1) “Your drinking pattern appears to fall within the ranges considered safe for most people your age and gender, and your results do not suggest that alcohol is harming your health” (“green light” feedback); 2) “Your results are below the range usually associated with harmful drinking or alcoholism, however, you may be at an increased risk for health problems due to the number of alcoholic drinks you reported consuming per week and or how much you have consumed on at least one occasion” (“yellow light” feedback); or 3) “It is likely that your current drinking patterns are hazardous or harmful to your health and well-being” (“red light” feedback). This was completed by patients after the computer was taken away, requiring them to have read and remembered what the computer displayed on the screen.

### Research staff experience and accurate deployment of CASI

Participating RAs were asked to complete a paper-and-pencil questionnaire at the end of every CASI administration to record in detail their experiences with implementing CASI. They recorded implementation barriers (logistical issues they encountered, such as internet/laptop/printer problems), study interruptions (e.g., patients needing to leave for an x-ray or to speak with physician, or patients needing a break because they weren’t feeling well), and patient comments (e.g., requests for CASI content clarification), and provided Likert ratings to quantify how much assistance they provided to each patient during CASI administration and how bothered the patient seemed while completing the study.

The RAs were also required to record which customized normative alcohol risk level handout they provided the patient to monitor fidelity to protocol and staff accuracy in disseminating correct educational take-home materials (low-, medium-, and high-risk alcohol use educational materials were preprinted, and RAs had to distribute the correct one).

### Validation of methods

#### CASI program validation

Prior to commencing the study, the lead author, in consultation with the data coordinating center, tested CASI’s scoring algorithm to ensure that patient’s alcohol use responses yielded accurate low-, medium-, and high-risk feedback profiles to the end user. No issues were identified during testing; feedback was accurate in all cases.

#### Protocol implementation validation procedures

At the beginning of the project, open-ended debriefing feedback was solicited from the first 10 patients and all five participating RAs to identify potential implementation barriers. Concerns related to three general themes need to be addressed to ensure the success of the study. These themes centered on sanitation, data security, and general technology concerns:

1. Sanitary concerns—One of the first patients provided excellent feedback, pointing out that some patients may be hesitant to enroll in a computer-based study because patients in the ED are mostly sick, perceived as contagious, and some patients may not want to touch a computer that was just used by someone else. We acted on this feedback by purchasing individual disposable ear-cover phone guards (http://www.northeasterntech.com) to go over the study headphones; purchasing a disinfectable medical-grade keyboard that was compliant for the hospital environment and marketed as being able to be sanitized in the dishwasher (http://www.man-machine.com/products/keyboards/really-cool-meditech.htm); and setting up a routine procedure where RAs would clean the laptop with a hospital-grade disinfectant in full view of the participants so patients would know it was cleaned between uses. We never received comments regarding sanitary concerns after these measures.

2. Equipment/data security—Research staff expressed concern about the potential of breaking or losing the tablet computer or its data. We acted on this feedback by working with the engineering department to locate a “designated home” for CASI equipment, properly tethered to the nurse’s station; purchasing a lock for the RA storage cabinet so the laptops could be stored in a locked cabinet during uncovered staff shifts; and purchasing a subscription to Computrace® lojack for laptops (http://www.lojack.com/pages/laptop.aspx) to increase data security. This device allows both remote data deletion and recovery of stored data in the event a computer is broken or stolen. With the implementation of these measures, initial hesitations about using computers for research in the ED were resolved. (As a point of interest, the laptop was never lost, stolen, broken or mishandled during this study).

3. General technology concerns—When the project began, research staff were generally hesitant about working on a computer-based project. Some of the RAs were not comfortable with computers themselves and had to be trained to troubleshoot simple internet signal loss and printing issues during the pilot phase. To increase the staff’s comfort level with CASI, individual training sessions were provided to RAs to go over computer troubleshooting and to answer any questions they had during the pilot phase. We also deputized a lead RA to answer questions from other RAs and to maintain a generally positive spirit about the importance of the research to other ED staff and RAs. An additional technology challenge in the first weeks of the study was difficulty maintaining wifi connectivity so the program could run properly. Instead of waiting for Information Technology (IT) department upgrades to reach the ED, we used a regular cell signal through a private cell carrier service, accessed via internet routers. Despite efforts to map out internet “dead” zones in the ED, the resulting spotty internet signal left us returning to the hospital’s IT network, which fortunately was available by that time. We learned early on that partnering with IT is very helpful in aiding ED staff who wants to set up a computerized program. Once IT became a member of the research team, technology issues became almost nonexistent. An alternate solution for other EDs wishing to implement a similar CASI program is to forego an internet-based program.

### Data analysis

Boston University served as the data coordinating center for CASI’s computer content, and all data were transmitted securely with data encryption over the internet to their site in real time. Data were anonymous but able to be merged based on matching ID numbers. At the conclusion of the study, the data were transferred to the principal investigator (PI) and merged with paper-and-pencil data. All paper data were then double-entered by a research secretary to ensure quality and minimize transcription error.

All data analyses were performed using SPSS, v 19. Descriptives were analyzed using means, ranges, and proportions (%) as appropriate to summarize and describe participant characteristics. Cross-tab procedures using Cohen’s kappa were used to assess the proportion of agreement between patients’ immediate recall of their alcohol-risk level (proxy for comprehension of alcohol-risk education) at the completion of the intervention and CASI’s calculation of alcohol-risk level. Cohen’s kappa was also used to assess the proportion of agreement between staff reports of which printed alcohol-education feedback they provided to each patient at the end of the intervention (staff accuracy) and CASI’s calculation of that patient’s alcohol risk level.

## Results

### Demographics

The median age of participants was 38 years (range, 21-85 years). Most of the sample was female and Hispanic, and the majority (86%) were being treated for less severe/complex medical conditions, as evidenced by nursing notes of emergency-severity index scores [23] of >3. Although offered in both English and Spanish, 90% of patients completed the program in English. Eighty percent of patients reported using a computer at home; of these, 72% reported using computers either daily or a few times per week, while 26% reported “rarely” or “never” using computers. See Table 1 for patient and ED visit characteristics.

**Table 1:** Patient characteristics (n = 517)

	**n (%)**
Sex (male)	191 (37)
Race/ethnicity	
Hispanic	321 (62)
Caucasian	35 (7)
African American	154 (30)
multiracial/other	11 (2)
Highest grade completed:	
elementary	25 (5)
some high school	102 (20)
high school or GED	156 (30)
some college or above	225 (44)
unknown	9 (1)
Employment:	
full time	266 (52)
part time	44 (9)
unemployed	134 (26)
disabled/retired/student/unknown	73 (13)
Computer experience:	
daily users	260 (50)
use few times per week	111 (22)
rarely use a computer	105 (20)
never uses computer	33 (6)
unknown/missing	8 (2)
Emergency Severity Index (ESI) triage level*	
≤2	49 (10)
≥3	436 (86)
unknown	32 (6)
Reason for visit category:	
pain	324 (63)
injury/accident	42 (8)
illness	68 (13)
other	79 (15)

*Score reflects acuity of patient’s chief compliant where higher numbers indicate lower acuity [23].

### Alcohol misuse

According to CASI alcohol screening results, 353 participants in this sample (68%) were low-risk drinkers, and 164 (32%) were at-risk drinkers; the latter included 123 (24%) who were medium-risk and 42 (8%) who were high-risk.

### Feasibility

#### Patient acceptance and comprehension

**Figure 1:**
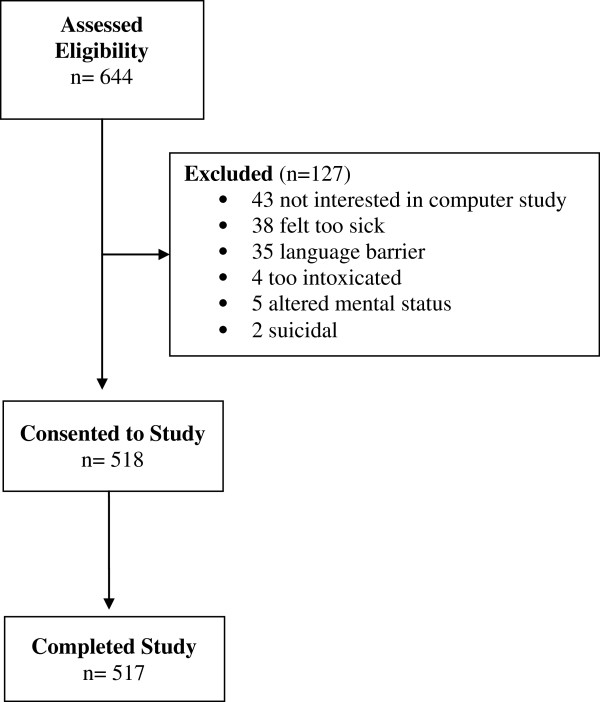
Study flow diagram.

Eighty percent of patients approached consented to participate (644 approached, 43 refused, 84 excluded as ineligible). Among patients who declined participation, primary reasons included not being interested in participating in a computer study (34%) and feeling too sick to participate (30%) (Figure 1). All but one patient who started CASI was able to complete the entire program (the very first patient was unable to sign back in after the internet signal was lost). No patients needed to take a break from the program because they did not feel well. The overwhelming majority (98%) of CASI administrations were completed without being interrupted by other clinical care activities. There were computer issues in 4% of the screenings for which an RA had to intervene; these included dropped a internet signal or printer problems. In all cases but one, patients were able to log back in and complete his or her data. The CASI program took an average of 15 minutes for patients to complete, although end times varied because patients had the choice of remaining on the website to review the alcohol education section, which many patients chose to do.

Ninety-seven percent of patients reported the language and content of the CASI program was respectful of them, 93% reported the program was easy to use, 93% reported feeling comfortable using a computer to receive this educational screening, and 89% reported liking the program (Table 2). Data revealed that patients accurately reported their alcohol risk (and therefore had comprehended their alcohol risk information) 90% of the time. Recall of alcohol risk-level feedback communicated immediately after the educational intervention revealed that 325 low-risk drinkers (94%), 98 medium-risk drinkers (83%), and 27 high-risk drinkers (79%) could correctly identify their alcohol risk level after participating in CASI program (kappa = 0.79) (Table 3).

**Table 2:** Patient feasibility and acceptance ratings of CASI

Feasibility Measures*	**n (% endorsed)**
Reported the program content was respectful of them	500 (97)
Reported the program was easy to use	483 (93)
Felt others would be helped by this program	479 (93)
Felt comfortable using computer to receive this education	478 (93)
Liked using the computer program	460 (89)
Found information useful to them	430 (83)
Would be willing to participate in future computer-based study during an emergency-department visit	409 (79)
Information learned got them thinking about their alcohol use	259 (50)
Learned something they would not have asked their doctor about	232 (45)
Reported some likelihood to change their alcohol use because of their study participation	198 (39)
Reported some intention to consult a health care professional about their alcohol use because of their study participation	144 (28)

*Data for 499 of 517 patients were available for analysis due to missing data. Totals represent dichotomous yes/no patient responses.

**Table 3:** Patient accuracy in comprehending alcohol risk level (n = 499)

**Feedback**	**n (%)**	**Kappa**
Computer calculated patient as low risk		
Patient self-report:		
- low risk	325 (94)*	
- medium risk	20 (6)	
- high risk	2 (1)	
		0.79
Computer calculated patient as medium risk		
Patient self-report:		
- low risk	8 (7)	
- medium risk	98 (83)*	
- high risk	12 (10)	
Computer calculated patient as High Risk		
Patient self-report:		
- low risk	6 (18)	
- medium risk	1 (3)	
- high risk	27 (79)*	

*Patient was accurate.

#### Research staff experiences and accurate deployment of CASI

Overall, research staff provided patients with the correct customized normative alcohol risk-level handout (take-home material) 97% of the time; 343 low-risk drinkers (97%), 113 medium-risk drinkers (99%), and 40 high-risk drinkers (93%) received the correct materials (kappa = 0.78) (Table 4). Staff reported in the majority of cases that patients did not appear bothered by completing the computer intervention, needed little to no assistance entering data, and sought few clarifications of words or content in the CASI program (Table 5).

**Table 4:** Staff accuracy in providing the correct educational feedback to patients to take home (n = 510)

**Feedback**	**n (%)**	**Kappa**
Computer calculated patient as low risk		
Staff provided:		
- low risk	343 (97)*	
- medium risk	9 (3)	
- high risk	1 (1)	
		0.78
Computer calculated patient as medium risk		
Staff provided:		
- low risk	0 (0)	
- medium risk	113 (99)*	
- high risk	1 (1)	
Computer calculated patient as high risk		
Staff provided:		
- low	2 (5)	
- medium Risk	1 (2)	
- high Risk	40 (93)*	

*Staff was accurate.

**Table 5:** Research staff experiences with CASI (n = 5) based on experiences with full sample (n=517)

**Feasibility Measures**	**n (% endorsed)**
Percentage of time research assistant (RA) rated they provided “very little” or “no” assistance to patients completing CASI	474 (92)
Percentage of time RA rated they felt the patient was bothered “very little” or “not at all” by using CASI	497 (97)
Patients who required RA to clarify meaning of CASI content	3 (<1)
Number of times CASI administration was interrupted by clinical care	4 (1)
Number of times staff needed to troubleshoot computer issues (e.g., printer problems)	6 (1)
Loss of Internet connection	21 (4)

## Discussion

The current study adds to a growing body of literature that finds ED-based computerized alcohol screening programs are both acceptable to patients and effective in educating them about their alcohol risk level. Our feasibility measures revealed that the majority of patients approached for study enrollment were willing to participate (80%), that patients rated the program as easy to use, and that they were not bothered about completing the survey during an unrelated ED visit. The CASI program accurately identified a high number of at-risk drinkers (32%). In addition to these positive findings, research staff reported few study interruptions and logistical barriers to implementing the CASI protocol.

Our measures of comprehension showed that the majority of patients (90%) accurately reported their alcohol risk level after participating in CASI, and, in 97% of cases, staff provided patients with correct alcohol-risk take home materials. Overall, these findings suggest that CASI facilitates efficient and accurate identification of at-risk drinkers and provides accurate alcohol-risk education to patients seeking care in the ED.

Regarding feasibility of recruitment, some patients chose not to enroll because they did not want to participate in a computer-based study. However, it should be noted that the majority or participants, even those who reported “rarely” or “never” using computers, were able to participate without difficulty. We were able to enroll large numbers of patients in a very short time (three months) from a diverse demographic pool. Anecdotally, RA staff reported that even elderly patients (one participant was 85 years old) and another with a broken dominant arm were still willing and able to complete the study without a problem. Furthermore, the study was conducted in the second largest ED in the United States, demonstrating that, even within the most busy and crowded ED settings, CASI can be successfully implemented.

Our findings lend support to Ranney et al’s [14] recent study, which concluded that there is a high preference for technology-based behavioral interventions among ED patients. In their survey of over 600 ED patients, the authors found a high baseline level of computer usage (91%), and 90% of their sample expressed a preference for a technology-based (versus face-to-face) intervention for at least one risk-behavior topic. Our results are also consistent with a recent systematic review of 20 technology-based SBIRT studies conducted among ED patients over the past 10 years that focused on high-risk health behaviors in the areas of alcohol/substance abuse, alcohol and youth violence, interpersonal violence, unintentional injury, mental health, and HIV risk [13]. These studies found high acceptance and feasibility for computer-based interventions among ED populations and supported their use in the ED to overcome the limitations of staff time, training, and resources, all of which hamper the feasibility of face-to-face ED interventions. They also highlighted the value of the sense of anonymity and privacy computer interventions can provide.

Our results are in contrast to Nilsen’s [16] recent study, however, which evaluated the implementation of a computerized alcohol-screening kiosk program among Swedish ED patients. In that study, only 41% of the target population completed their alcohol survey when directed to do so by the waiting-room triage nurse upon arrival in the ED. The authors cited various logistical barriers (staff, patient health, etc.) resulting in low participation. It should also be noted that the model used in the Nilsen study called for “minimal researcher involvement to test the concept’s viability under realistic conditions,” and the program was unattended by any dedicated staff. The lack of oversight of the project (beyond a nurse handing patients index cards instructing them to complete the test and someone checking the printer to make sure it had paper to print out results) was the most likely contributor to this study’s low acceptance and participation rates. Although laudable for attempting to implement a stand-alone program with little oversight, it is likely that computer-screening programs require some degree of staff involvement and a staff “champion” to be successful.

An additional topic highlighted by the current study is the need to focus on patient comprehension to ensure effective knowledge transfer within educational interventions. To measure the feasibility of implementing an ED-based SBIRT program, we felt it was critical to assess the patient’s comprehension of the educational materials CASI provided. We are not aware of any other ED studies that focused on comprehension as a key outcome. However, with the growing problem of health illiteracy in the US and the fact that many patients who use the ED have low health literacy [24], it is critical that interventions begin with this step during the developmental phase. This often-overlooked step must be done to ensure patients are absorbing the educational content provided during and ED intervention. This seems especially important when one considers that patients are often feeling very ill and are likely distracted by their current health status in the middle of an ED visit. Although it was only possible to use an immediate and cued recall test within the confines of this pilot study as a proxy for comprehension (and patients were presented with the computer-screening results within a few minutes of being asked to recall it), we feel it provides clear and objective evidence that, at a minimum, patients were able to read and retain the health information provided to them during the CASI intervention. Although this is an important first step, future studies would do well to use a more comprehensive measure of comprehension to be sure patients understand the meaning of their alcohol-risk levels.

Regarding comprehension, these feasibility data also provide objective criteria for crafting more comprehensible and comprehensive health education messages for future studies. For example, we found that patients in this study did not comprehend high or medium alcohol-risk education as well as the low-risk education (79%, 83%, and 94%, respectively). This may have been due in part to patients choosing not to attend to a “message they do not want to remember” versus true lack of recall or comprehension. However, with a comprehension discrepancy as high as 15% (low- versus high-risk categories), and based on close scrutiny of the length and grade level of the educational feedback provided, this finding also suggests the health message used for the medium- and high-risk groups may have been less well-understood, and thus may warrant revision.

Additionally, some feasibility/acceptance measures used in this study were endorsed below the 50% level. However, all endorsements would be considered clinically meaningful and desirable outcomes. For example, although only 28% of the patients said they had “some intention to consult a health-care professional about their alcohol use because of their study participation,” it is highly desirable to find 28% of patients recognizing the need to speak with a professional about their alcohol use. This percentage is also very close to the percentage of “at-risk” alcohol users (32%) found in the study.

A secondary goal of the project was to measure accurate deployment of alcohol risk-level education to patients. Our data reveal that, in a few cases, RAs did not provide patients with the correct take-home alcohol-risk materials. Nevertheless, the correct information did appear on the patient’s computer screen, and *all* patients received the same general alcohol education pamphlet, including NIAAA low-risk drinking guidelines and an 800 number to contact a treatment locator hotline. Therefore, although the correct take-home educational materials were not accurately deployed to every patient, no patients received “bad” take-home information, and the computer providing accurate information consistently and without fail.

Regarding limitations, the current study was limited by a cross-sectional design and use of a convenience sample. Additionally, we did not use a comparator group or follow-up with patients to see if they acted on the alcohol risk-level education provided. There were also fewer men and white patients in our sample, so gender and racial differences could not be fully explored. In addition, we were unable to modify the computer content because it was provided free to us by another website. As always, self-reporting bias is an inherent problem in studies of this nature. However, we feel that the anonymous and confidential manner in which patients were able to report their alcohol use likely minimized social undesirability and under-reporting of alcohol use in this study.

## Conclusions

This study demonstrates that computer-delievered brief alcohol screening and intervention programs can be successfully implemented within a busy ED setting, are acceptable to most patients, and can identify a high number of ED patients with at-risk drinking. Additionally, this study demonstrates that few logistical problems related to using computers for these interventions were experienced by patients or research staff. Further studies including a control group and follow-up measures are needed to evaluate the CASI program and determine whether it can reduce alcohol use post-intervention.

## Competing interests

The authors declare that they have no competing interests.

## Authors’ contributions

MM developed the study question, protocol, trained and supervised all research staff, served as Principal Investigator supervising the conduct of the study, drafted the manuscript and integrated all co-authors contributions. PB contributed to the research design and conducted all statistical analyses in close collaboration with MM. DR facilitated the oversight of the data coordinating center at Boston University. SB contributed to research design and provided guidance on implementing an SBIRT protocol in the ED environment. EJG contributed research design expertise and choice of outcome measures. All authors read and approved the final manuscript.

## Disclosures

This study was supported by funding from Join Together (Boston University) and the Bronx Center to Reduce and Eliminate Ethnic and Racial Health Disparities (Bronx CREED). These results were presented in part at the annual meeting of the Society for Academic Emergency Medicine, Boston MA, June 2011.
